# Notes and News

**Published:** 1903-04-18

**Authors:** 


### Notes and News.

On April 27th, his Royal Highness the Prince of Wales will lay the foundation stone of the new workmen's dwellings to be erected in Regency Street, Westminster; the ceremony is to be followed by a Reception by the Mayor and Mayoress of Westminster at the Caxton Hall.

His Royal Highness the Prince of Wales has consented to take the chair at the festival dinner on June 10th in aid of the jubilee fund for the pensioners and foundation scholars of the Royal Medical Benevolent College. The institution benefits a class which has especial claims upon public benevolence, for it helps the orphans of medical men and gives assistance to members of the profession who are past work, or to their widows who have no prospect before them but the workhouse. Pensions of £30 a year are given to 50 aged medical men or to their widows, while 50 sons of medical men are provided with a first-class education, with clothing and maintenance. An appeal is now being made to the public for the sum of £10,000, which is required to secure the continuance of this charitable work unimpaired.

A drawing-room meeting was held last week at Stafford House by the kind permission of the Duke and Duchess of Sutherland, on behalf of the Field Lane Ragged Schools and Refuges. The institution was founded in 1841. Amongst other things it includes a crêche for babies, an industrial home for boys, an institute for young men, a club for young women, and a Band of Hope and temperance society. It requires nearly £8,000 per annum to support this good work and upwards of £4,500 has to be raised by voluntary contributions. During the past year 280 people had been placed in or assisted to obtain employment, 150 boys and girls were maintained in industrial homes and there had been 49,022 attendances at the Bible ragged schools and classes. Many other interesting statistics were shown.

The King's College Hospital Banquet will take place in the Whitehall Rooms, Hôtel Métropole, on April 28th.

The Lord Mayor will preside at the banquet which will be held on April 30th at the Hôtel Métropole on behalf of the National Hospital for the Paralysed and Epileptic.

The Prince and Princess of Wales (grand president and lady grand president) have been pleased to appoint the following presidents and lady presidents of the League of Mercy :—The Earl Cadogan, president of the Chelsea district; the Earl of Kilmorey, president of the Westminster district; Dora Countess of Chesterfield, lady president of the Kensington (South) district; Lord Hugh Cecil, M.P., president of the Greenwich district; Lady Somerset, lady president, and Mr. Lyons Walcott, president of the Enfield district; General Sir William Stirling-Hamilton and Lady Stirling-Hamilton, president and lady president of the Horsham district; Mr. Leopold Rothschild and Mrs. Rothschild, president and lady president of the Ealing district; Mr. Charles Henry and Mrs. Henry, president and lady president of the Paddington (North) district; Dr. Scott, president of the Islington (West) district; the Rev. H. Russell Wakefield, president of the Marylebone (East) district; and Mrs. Lefroy, lady president of the Harwich district of the League of Mercy.

The new scheme for the management of Addenbrooke's Hospital, Cambridge, which has been referred to in previous numbers of The Hospital, has now been confirmed by Parliament. The main feature of the scheme is the establishment of a general committee of management to carry on the affairs of the hospital, which duties, by the Act of 1766, have up to the present, rested with the governors at large, who now number about 600. The members of the general committee are to be elected by the governors in general court, eight being chosen from residents or ratepayers in the borough, eight from residents or ratepayers outside the borough, and eight from the electoral roll of the University, making 24 members in all. One half of the committee is to retire annually, the retiring members being eligible for re-election. The honorary medical and surgical staff may nominate two members of their body who can be present at all meetings of the general committee as advisers. The duties of the general committee are to conduct and carry on the entire administration of the hospital, subject only to certain reservations where their proceedings are subject to confirmation by a general court of governors, such as the sale of any securities on behalf of the hospital, or the election and dismissal of members of the staff. The general committee is to meet once in each week at the hospital for the despatch of business. Seven members will form a quorum on the first weekly meeting in each month and at any specially summoned meeting; at all other times three shall be the quorum. It is very satisfactory to feel that the management of Addenbrooke's Hospital is now permanently established on principles which are both practical and up to date.

